# High-performance Collective Biomarker from Liquid Biopsy for Diagnosis of Pancreatic Cancer Based on Mass Spectrometry and Machine Learning

**DOI:** 10.7150/jca.63244

**Published:** 2021-11-04

**Authors:** Tomohiko Iwano, Kentaro Yoshimura, Genki Watanabe, Ryo Saito, Sho Kiritani, Hiromichi Kawaida, Takeshi Moriguchi, Tasuku Murata, Koretsugu Ogata, Daisuke Ichikawa, Junichi Arita, Kiyoshi Hasegawa, Sen Takeda

**Affiliations:** 1 Department of Anatomy and Cell Biology, Faculty of Medicine, University of Yamanashi, Chuo, Yamanashi, Japan; 2 Hepato-Biliary-Pancreatic Surgery Division, Department of Surgery, Graduate School of Medicine, The University of Tokyo, Tokyo, Japan; 3 First Department of Surgery, Faculty of Medicine, University of Yamanashi, Chuo, Yamanashi, Japan; 4 Department of Emergency and Critical Care Medicine, Faculty of Medicine, University of Yamanashi, Chuo, Yamanashi, Japan; 5 Shimadzu Corporation, Nakagyo, Kyoto, Japan

**Keywords:** Pancreatic ductal adenocarcinoma, Liquid biopsy, Metabolome, Machine leargning, Neoadjuvant chemotherapy

## Abstract

**Background:** Most pancreatic cancers are found at progressive stages when they cannot be surgically removed. Therefore, a highly accurate early detection method is urgently needed.

**Methods:** This study analyzed serum from Japanese patients who suffered from pancreatic ductal adenocarcinoma (PDAC) and aimed to establish a PDAC-diagnostic system with metabolites in serum. Two groups of metabolites, primary metabolites (PM) and phospholipids (PL), were analyzed using liquid chromatography/electrospray ionization mass spectrometry. A support vector machine was employed to establish a machine learning-based diagnostic algorithm.

**Results:** Integrating PM and PL databases improved cancer diagnostic accuracy and the area under the receiver operating characteristic curve. It was more effective than the algorithm based on either PM or PL database, or single metabolites as a biomarker. Subsequently, 36 statistically significant metabolites were fed into the algorithm as a collective biomarker, which improved results by accomplishing 97.4% and was further validated by additional serum. Interestingly, specific clusters of metabolites from patients with preoperative neoadjuvant chemotherapy (NAC) showed different patterns from those without NAC and were somewhat comparable to those of the control.

**Conclusion:** We propose an efficient screening system for PDAC with high accuracy by liquid biopsy and potential biomarkers useful for assessing NAC performance.

## Introduction

An increasing number of patients have pancreatic cancer, which is currently the fourth leading cause of annual mortality of almost 35,000 in Japan 1. Moreover, pancreatic ductal adenocarcinoma (PDAC) accounts for around 90% of malignant pancreatic tumors. The only curable treatment for pancreatic cancers is surgical tumor resection, although >80% of cases are at unresectable stages 2, 3. Therefore, screening strategies ideal for detecting curable PDAC are awaited. Classic tumor markers such as carbohydrate antigen 19-9 (CA19-9) and carcinoembryonic antigen are routinely used for screening and detecting PDAC. However, the relatively low true positivity in the early stages prevents tumor detection at a curable state 4-6. Alternative imaging modalities such as ultrasound scan, computed tomography (CT), and magnetic resonance cholangiopancreatography (MRCP) are incapable of delineating the tumor with a small diameter 7. With all those in mind, a new strategy revolutionizing pancreatic cancer diagnostics is needed.

An avalanche of studies has used biopsy specimens taken by surgery or endoscopic ultrasound-fine needle aspiration. However, bona fide heterogeneity in tumors makes it difficult to look for an ideal biomarker. Furthermore, the repetitive and time-consuming examination renders patients reluctant to accept the clinical trial 8, 9. Considering the availability of body fluids, the so-called liquid biopsy, including plasma, serum, and urine, has been intensively investigated to meet the clinical needs and patients' benefit by useful biomarkers for pancreatic cancer 10-15.

Genetic mutations, either in proto-oncogenes or tumor suppressor genes 16, incur some metabolic changes in tumor cells. Therefore, downstream metabolites showing patterns reflecting the metabolic changes due to genetic mutation, either directly or indirectly, are reasonably assumed 17. Along with this notion, mass spectrometry (MS) analyzes the metabolites (e.g., phospholipid (PL), primary metabolite (PM), and lipid mediators). Several groups reported specific biomarkers for pancreatic cancer from serum by making most of the Fourier-transform ion cyclotron resonance MS 18, gas chromatography MS 19, 20, and liquid chromatography/electrospray ionization MS (LC/ESI-MS)^21^-^23^.

We have developed a medical device based on MS and machine learning and validated the performance in diagnosing various diseases. For example, the current prototype apparatus equipped with probe electrospray ionization-MS (PESI-MS) 24 is superior to other MS-based instruments 25 in clinical settings due to procedure simplicity, analysis rapidity, and cost performance 26, 27. This instrumentation enables the discrimination of diseases from the background (e.g., head and neck tumor and breast cancer) 28-31. Recently, the serum data that this study system analyzed predicted PDAC with sensitivity and specificity superior to CA19-9 32. This successful prediction supports the idea that body fluid is an appropriate human sample to establish a novel method for detecting early PDAC. The PESI-MS system gives preference to the sample preparation simplicity and provides a metabolome fingerprint with >2,000 variables but does not target specific molecules. However, too many variables sometimes cause overfitting to a specific sample group or is disturbed by background noise, as summarized in previous reviews 33, 34. Therefore, targeted metabolite selection is an option for algorithm optimization. For targeting, LC/ESI-MS is a method to analyze and annotate specific molecules.

A collective biomarker, considering the unique combination of PM and PL, has not been employed yet, while LC/ESI-MS has been applied to discover metabolites specific to the pancreatic tumor 19, 22, 35, 36. This study analyzed the serum with LC/ESI-MS to obtain metabolome and established a diagnostic algorithm specific to pancreatic cancer on PM and PL databases. A higher diagnostic accuracy has been achieved compared with other previous techniques. Interestingly, the current method suggests the monitoring capability of the metabolic changes in neoadjuvant chemotherapy (NAC)-treated patients reflecting the effectiveness of preoperative chemotherapy. While NAC was recently incorporated into the therapeutic procedure for PDAC for prognosis improvement, it is generally difficult to evaluate NAC's effects even with the combination of CA19-9 and imaging diagnosis. Thus, the current strategy can pave a new way in NAC evaluation in clinical settings by liquid biopsy.

## Methods

### Ethics statement

This study followed the ethical standards of the Declaration of Helsinki. We fractioned serum samples from Japanese patients who underwent pancreatic cancer surgery at the University of Yamanashi Hospital or the University of Tokyo Hospital. This study used serum from outpatients transported to the University of Yamanashi Hospital's emergency department as the control. The ethics committee of the University of Yamanashi (nos. 2086 and 2220) or the University of Tokyo (no. 2019370NI) approved the study protocol. Patients provided informed consent in a written format with opt-out. Pathological diagnosis was according to the Union for International Cancer Control (UICC) TNM classification of malignant tumors. All clinical data were anonymized.

### Neoadjuvant chemotherapy

The regimens of neoadjuvant chemotherapy (NAC) were Gemcitabine/S-1 (GS) or Gemcitabine/nab-Paclitaxel (GnP). The efficacy of patients with NAC was summarized in Table S1.

### Inclusion and exclusion criteria

Preoperative serum was obtained from patients before receiving surgical resection. This study enrolled 77 cases (37 from the University of Yamanashi Hospital and 40 from the University of Tokyo Hospital). Out of them, this study used 29 cases whose cancer grades of T2 or T3 according to the TNM classification (Fig. S1). Moreover, 30 cases of control serum were obtained from the outpatients transported to the emergency department, who had not been admitted to the hospital. The patients were 56-87 years old. Furthermore, those with a medical history of cancer, diabetes, dyslipidemia, and chronic kidney disease were not included in the control group (Table S2).

### Clinical sample preparation

For PL analysis, 990 µL of 0.1% formic acid in methanol were mixed with 10 µL of serum by ThermoMixer C (Eppendorf, CA, USA) for 5 min at 4 °C. The sample solution was centrifuged at 15,000×g for 10 min after being incubated on ice for 10 min. The resultant supernatant was twofold diluted using 0.1% formic acid in methanol, from which 300 µL diluted supernatant was placed into a LabTotal Vial (Shimadzu Corporation, Kyoto, Japan). Moreover, 50 µL serum was mixed with 500 µL of 0.1% formic acid in methanol to analyze PM. After adding 250 µL of pure water, 500 µL chloroform was sequentially placed, followed by vortexing for 10 min. Subsequently, the mixture was centrifuged at 15,000×g for 10 min. Also, 400 µL of the upper layer was taken and applied to Amicon Ultra 0.5 mL centrifugal filter MWCO 3 kDa (MERCK KGAA, Darmstadt, Germany). The filtrate was dried up by a centrifugal vacuum concentrator (TAITEC Corporation, Saitama, Japan). The resultant components were redissolved into 200 µL pure water. Finally, the sample was applied to TORAST-H Bio Vial (Shimadzu Corporation).

### Mass spectrometry

LC/ESI-MS was performed using the high-pressure LC installed LCMS-8060 (Shimadzu Corporation) system. The LC/MS/MS Method Package for Phospholipid Profiling (Shimadzu Corporation) was used according to the manufacturer's instructions to analyze the PL. Kinetex C8 column (Kinetex C8, 150 mm × 2.1 mm i.d., 3.6 μm particle size; Phenomenex, Torrance, CA, USA) with mobile phases A (20 mM ammonium formate in water) and B (acetonitrile-isopropanol 1:1 *v*/*v*) were employed for LC separation. The mobile phase B gradient was programmed as 20% (0 min)-20% (1 min)-40% (2 min)-92.5% (25 min). The column oven temperature was 45 °C. To analyze the PM, LC/MS/MS Method Package for Primary Metabolites, Ver. 2, was used. Discovery HS F5-3 column (150 mm × 2.1 mm I.D. ×, 3 µm particle size; Merk & Co., Kenilworth, NJ, USA) with mobile phases A (0.1% formic acid in water) and B (0.1% formic acid in acetonitrile) was used for LC separation. Furthermore, mobile phase B gradient was programmed as 0% (0 min)-25% (51 min)-35% (11 min)-95% (20 min). The column oven temperature changed to 40 °C.

### Statistical analysis

Data processing and molecular identification/quantification were performed automatically by LabSolutions (version 5.82 SP1; Shimadzu Corporation). Obtained data were stored in a relational database with clinical parameters. For statistical analysis, we normalized mass spectra using a median with autoscaling for each metabolite. Statistical and biomarker analyses performed partial least square regression (PLSR) analysis, heatmap, and receiver operating characteristic (ROC) curve on MetaboAnalyst 5.0 (https://www.metaboanalyst.ca/). We drew ROC curves on the probabilities calculated using the support vector machine (SVM) based on each group's ion intensities. Cancer probability was shown as an average of 100 runs of Monte Carlo cross-validation using two-thirds of the total samples. The metabolite ratio was calculated using normalized ion intensity values in LC/ESI-MS between cancer and control groups. *P*-values were calculated using the Student's *t*-test (for two groups) and one-way analysis of variance (ANOVA) (for three groups) to show the statistical significance of specific molecules. Moreover, Microsoft Excel and GraphPad Prism 7.0 were used for arithmetic and graphs. We analyzed the metabolomics pathway with the normalized metabolome data by median and autoscaling on MetaboAnalyst 5.0.

## Results

### Patients' characteristics

The clinical PDAC characteristics and control patient cohort are summarized in Table 1. This study collected serum samples from 77 patients who underwent pancreatic cancer operation at the University of Tokyo Hospital or the University of Yamanashi Hospital from November 2019 to October 2020. To focus on the PDAC at progressed but operable stage, we selected 29 samples at stage T2 and T3 (T as for the TMN classification) (Fig. S1). Of the 29 samples, we used 19 with earlier sampling dates (November 2019 to July 2020) for database construction (training samples). The remaining 10 samples collected after August 2020 were used as the validation cohort (validation samples; Table 1). Moreover, 30 patients from the cancer-free cohort were divided into 20 and 10 for database construction and validation, respectively, based on sample splitting according to the collection date.

### Diagnostic algorithm construction

**Figure 1:**
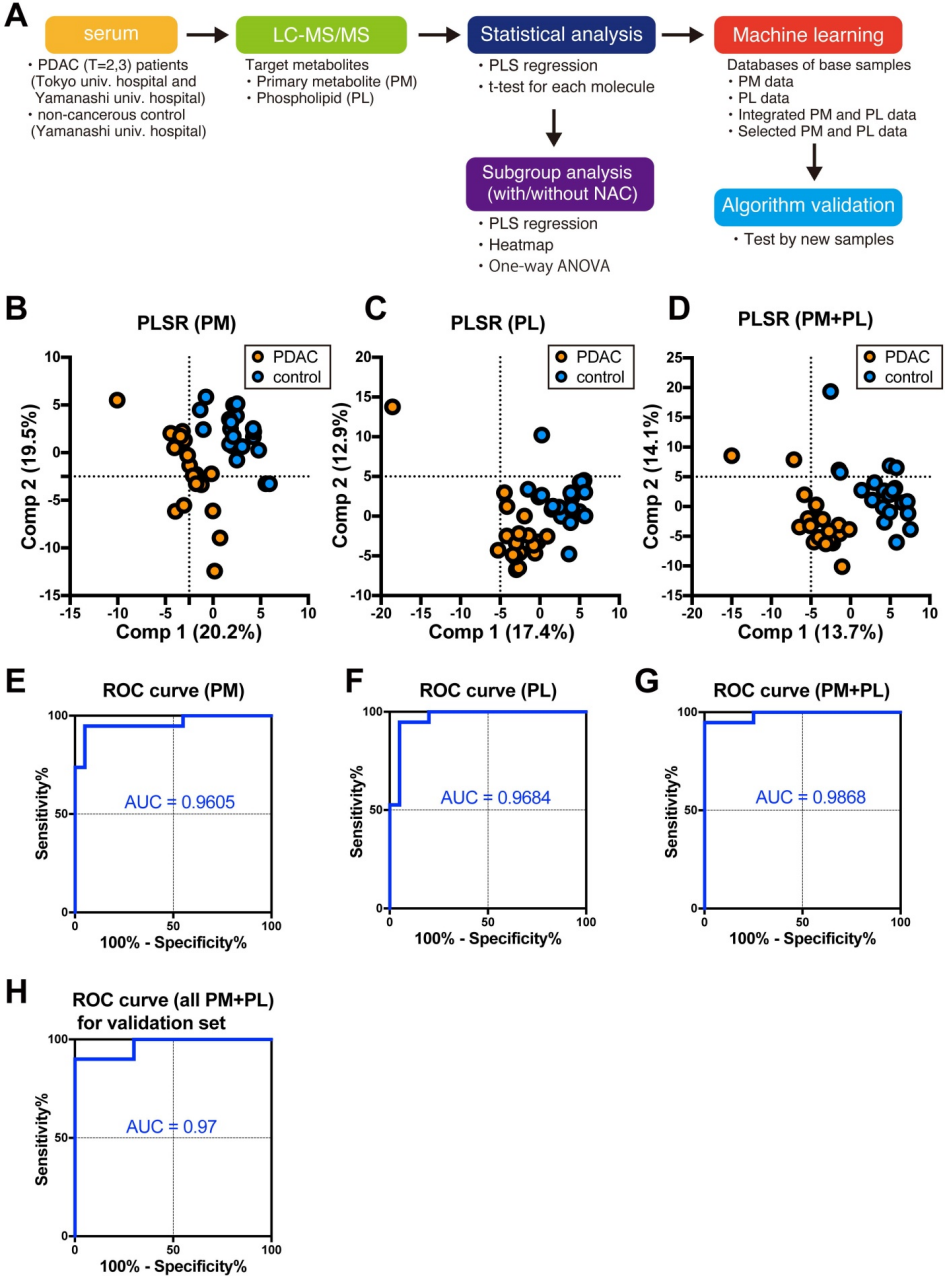
** The analysis flow and diagnostic outcomes by machine learning. A** The design and flow of this study. **B-D** Scatter plots of PLSR for PDAC and control by metabolome data. *Orange* and *blue plots* indicate PDAC and control, respectively. Three sets of a database composed of 91 PMs (**B**), 178 PLs (**C**), and a combination of both (**D**) were used. A variance of each principal component (*Comp*) is indicated on the ordinate and abscissa. **E-H** ROC curves were drawn with sensitivity on the ordinate false-positive fraction on the abscissa. Support vector machine was fed with the same database used for PLSR as shown in **B**-**D**. Validation of the independent cohort gave similar outcomes as shown in **G**. *PM* primary metabolites, *PL* phospholipids, *PDAC* pancreatic ductal adenocarcinoma, *PLSR* partial least square regression, *ROC* receiver operating characteristics, *AUC* area under the curve, *SVM* support vector machine.

This study first analyzed 39 serum collected from either PDAC patients (*n* = 19) or control cohort (*n* = 20) with two independent method packages for either PM or PL by LC/ESI-MS. Of the 97 PMs and 422 PLs in the targeted metabolites registered in the method packages, 91 PMs and 178 PLs were identified and successfully quantified. Three separate databases were composed of ion intensities of either identified PM or PL, and integrated PM and PL data (Fig. 1A). PLSR discriminates PDAC cases from the control group by any databases (Fig. 1B-D). Furthermore, we fed each normalized ion intensity of these trainig samples into the database for SVM, a machine learning, to test if the algorithm can distinguish PDAC from control cases. Sensitivity, specificity, and accuracy were calculated based on the SVM outcome in terms of concordance ratio. All other parameters exceeded 90% except for specificity and accuracy based on the PM, suggesting the higher potential for integrated PM and PL data in predicting the PDAC (Table 2). ROC curves elegantly indicate this strategy's high performance (Fig. 1E-G). Moreover, the area under the curve (AUC) was 0.9605, 0.9684, and 0.9868 for PM, PL, and their combination, respectively (Table 2).

This model was further validated by an independent cohort not included in the training samples (Table 1). Sensitivity, specificity, and accuracy were 90%, 100%, and 95%, respectively, for validation samples containing 20 cases (Table 2). AUC was 0.97 (Fig. 1H and Table 2). Thus, these results strongly support that the current diagnostic system based on the PM and PL integrated database can distinguish PDAC serum from control.

### Molecular cluster optimization by machine learning database for PDAC diagnosis

**Figure 2:**
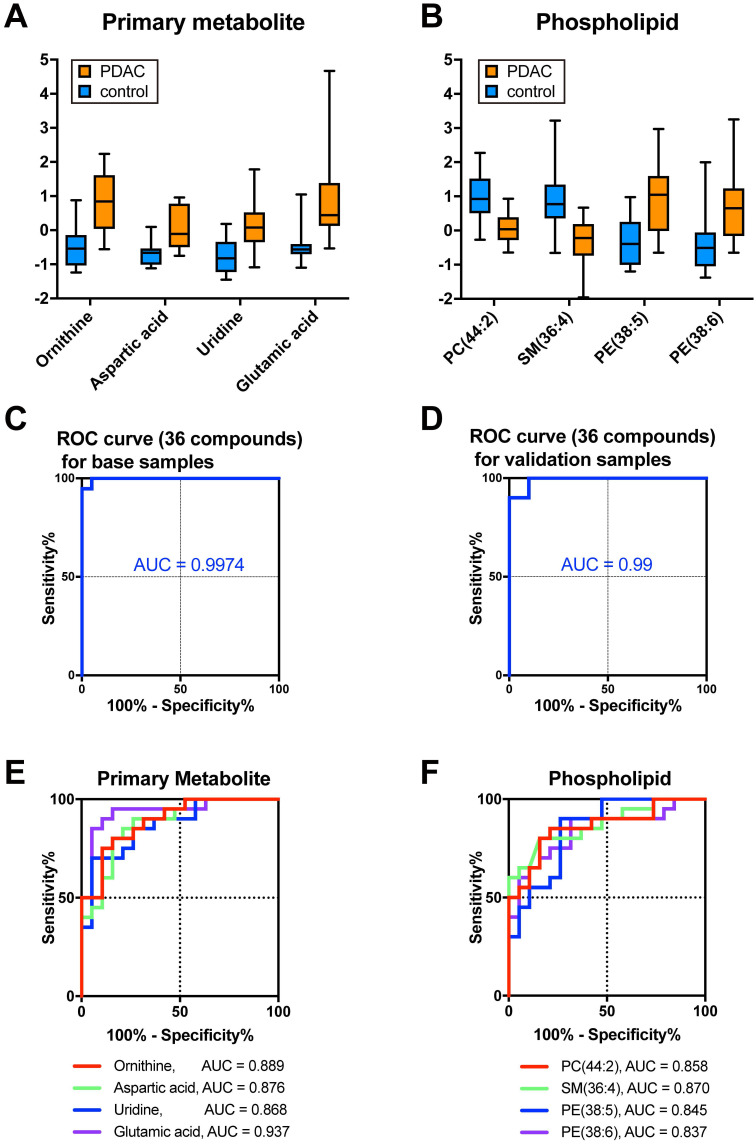
** The database based on the selected PM and PL (collective biomarker) gave the strongest prediction for PDAC than a single biomarker. A, B** *Boxplots* of representative primary metabolites **(A)** and phospholipids **(B)** significantly changed in PDAC. Ion intensities were normalized by median and autoscaling with MetaboAnalyst 5.0. C, D ROC curves were drawn based on the database with selected 36 metabolites **(C)** from the training dataset or 36 metabolites from the validation dataset **(D)**. E, F ROC curves were drawn for each primary metabolite **(E)** and phospholipid **(F)**. AUC was calculated from the ROC curves. *PM* primary metabolites, *PL* phospholipids, *PDAC* pancreatic ductal adenocarcinoma, *ROC* receiver operating characteristics, *AUC* area under the curve.

The selection of variable factors (metabolites) is important to optimize the algorithm as summarized in previous reviews 33, 34. Too many variables sometimes cause overfitting to one side or fitting to noise data. This study then focused on the specific metabolites whose expression changed significantly in PDAC. To narrow down the candidate molecules responsible for discrimination, we used *p*-values <0.03 (>1.523 in -log10 transformation) and absolute fold-change >0.6 in log2 transformation. According to this criteria, 15 PMs and 21 PLs were screened (Table 3). Moreover, most of the molecules were upregulated in PDAC in the case of PM (Fig. 2A and Table 3). This study took advantage of the MetaboAnalyst and obtained several metabolic cascade candidates to identify which metabolites contribute to the metabolic pathway emphasized in PDAC (Fig. S2A and Table S3). For example, the arginine biosynthesis pathway activation can cause PDAC metabolic changes taking account of a series of significantly upregulated molecular species (e.g., ornithine, aspartic acid, glutamic acid, and arginine; Fig. S2B). Concerning PL, both phosphatidylcholine (PC), lysophosphatidylcholine (LPC) and sphingomyelin levels decreased in PDAC, while phosphatidylethanolamine (PE), and lysophosphatidylethanolamine (LPE) increased in PDAC (Fig. 2B and Table 3). Sensitivity, specificity, and accuracy in training 39 samples were 100%, 95.0%, and 97.4%, respectively, with an AUC of 0.9974, when 36 PM and PL were selectively assembled as a new database (Fig. 2C and Table 2). To assess the algorithm's power, we used 20 validation samples, where sensitivity, specificity, and accuracy were 90%, 100%, and 95%, respectively (Table 2) with an AUC of 0.99 (Fig. 2D and Table 2). Therefore, AUC exceeded those of any molecule with significantly lower *p*-values (e.g., ornithine, aspartic acid, uridine, or glutamic acid; Fig. 2E), and PC (44:2), SM (36:4), PE (38:5), and PE (38:6; Fig. 2F). The most efficient diagnostic process should be based on the specific PM and PL data, while the molecular background is still an open question.

### Bipartite changes in metabolic serum profiles by neoadjuvant therapy

**Figure 3:**
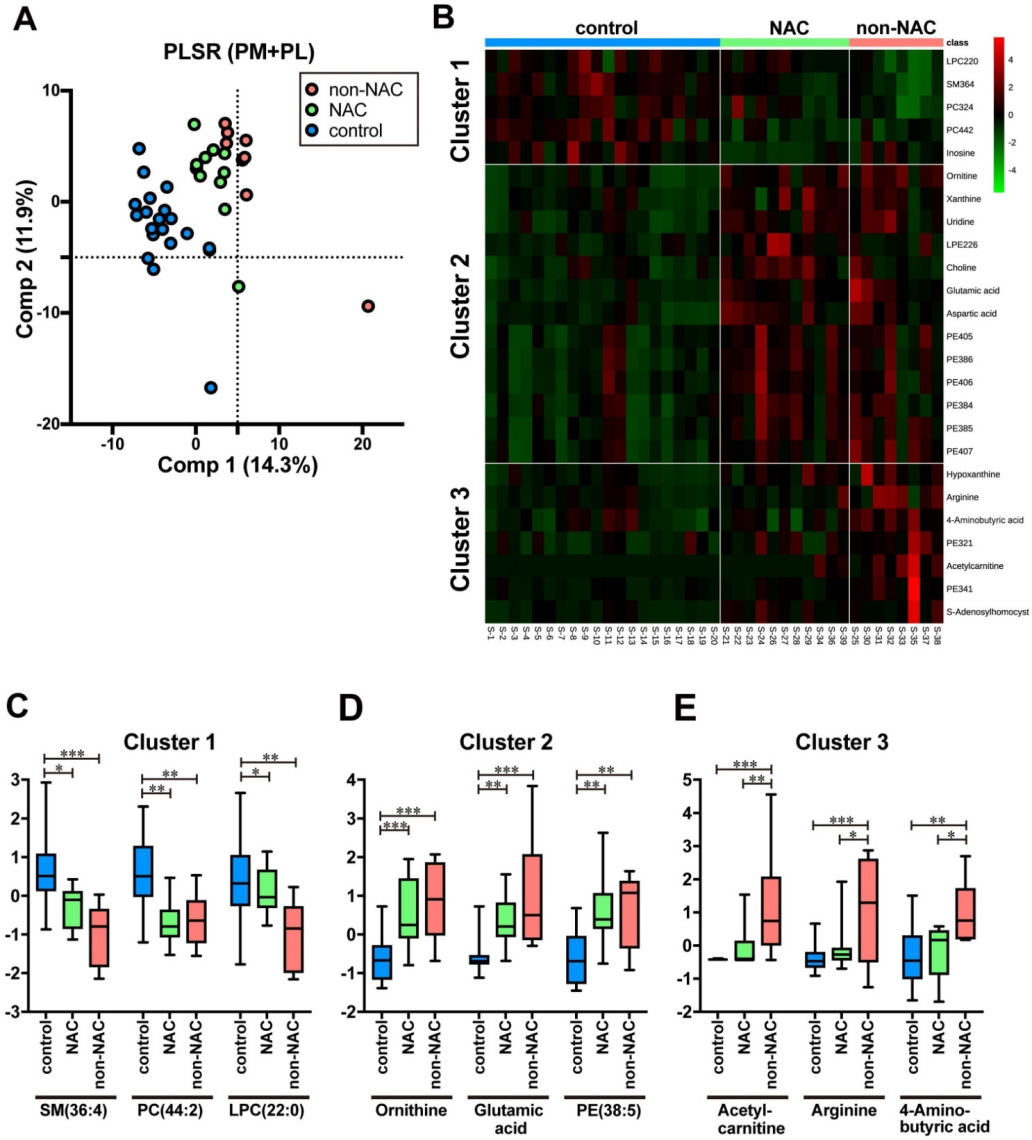
** NAC treatment affected the metabolic profiles of the patients' serum. A** *Scatterplots* of PLSR of PDAC without NAC (non-NAC), with NAC (NAC), and control by metabolome data. *Pink*, *green*, and *blue* plots indicate non-NAC, NAC, and control, respectively. Database with integrated 269 PM and PL were used. The variance of each principal component (*Comp*) is indicated on the ordinate and abscissa. **B** Heatmap showing the normalized ion intensity of significantly differed metabolites for each group. Metabolites with similar ion intensity patterns were arbitrarily assembled into three clusters. **C**-**E** *Boxplots* of metabolites significantly representing each cluster are shown. Ion intensity of each spectrum was normalized with median and autoscaling by MetaboAnalyst 5.0. *PDAC* pancreatic ductal adenocarcinoma, *NAC* neoadjuvant chemotherapy, *PLSR* partial least square regression, *PM* primary metabolites, *PL* phospholipids. Statistical significance was assigned *p < 0.05, **p < 0.01, and ***p <0.001.

In this study, 20 out of 29 cases have gone through NAC (Table 1 and Table S1). First, three groups were constructed comprising PDAC without NAC (*n* = 8, non-NAC), PDAC with NAC (*n* = 11, NAC), and control (*n* = 20) in the training samples to evaluate the effects of NAC on the metabolites identified in the previous section. Three groups are separated with minimal PLSR overlap (Fig. 3A). Intriguingly, clustered NAC plots located between those of the control and non-NAC imply that NAC brought about metabolic changes that rectify PDAC-specific profile to control (Fig. 3A). We prepared a heat map and identified three different PM and PL clusters, which reflected each serum's status to delineate each metabolite's differences more explicitly (Fig. 3B). On the one hand, cluster 1 is chiefly composed of PL, such as SM (36:4), PC (44:2), and LPC (22:0). Moreover, most of them were predominantly detected in control (Fig 3C). On the other hand, cluster 2 comprises PM (e.g., ornithine, glutamic acid) and PL, such as PE (38:5). They were mostly upregulated in PDAC serum regardless of NAC (Fig 3D). Therefore, the molecular expression belonging to cluster 2 may be an impregnation of PDAC to serum. Cluster 3 is strongly upregulated in non-NAC, witnessing the authentic PDAC state. Acetylcarnitine, arginine, and 4-aminobutyric acid are representative of cluster 3 (Fig 3E). Moreover, the NAC group's serum took a unique position, which shared the metabolic profiles of both control and PDAC. Currently, neither PM nor PL species, specific to the NAC samples, have been identified yet.

## Discussion

### High-performance collective biomarker for PDAC prediction

This study addressed the issue crucial for PDAC diagnosis and evaluation of the effect of NAC. We evaluated the predictive power for machine learning-based diagnosis system of PDAC using a metabolite database from the Japanese cohort through ambient mass spectrometry. Technically, this is based on the ion intensities of the primary metabolites and phospholipids, which are annotated and quantified by LC/ESI-MS. The predictive power should be emphasized as the strongest when significantly differed metabolites, 15 PM and 21 PL, were confined into the database for machine learning rather than exhaustive registration of the whole data into the database. Furthermore, the predictive power of machine learning with the selected metabolites was superior to any single molecule. Therefore, the term *collective biomarker* refers to this molecule cluster. Higher performance in PDAC prediction by the collective biomarker was accomplished and was only applicable to PDAC-specific stages (T factors 2 and 3). Although the database is based on the training samples that largely include higher T (T3: 84%) and N (*N* > 0, 68%) factors with UICC stage II, the current system can also discriminate the validation of cancerous samples that mainly consist of the lower T and N factors (T2, 60%; N0, 60%) with the lower UICC stage IB. This strongly suggests that the current database harbours the PDAC nature and can be applied to the discrimination of the lower stages. Specifying and adding more candidate molecules for machine learning is needed to broaden the current system's applicability to PDAC diagnosis, in general.

### Biochemical pathway altered in PDAC

Several metabolites that feature PDAC in Japanese cohorts were identified. While some phospholipids (e.g., LPC (18:2), PE (38:5), and SM (36:4)) have been previously reported as the PDAC-featuring metabolites 18, 37, a couple of newly identified specific metabolites in PDAC serum were added.

Ornithine, aspartic acid, glutamic acid, and arginine all belong to the arginine synthesis pathway, and are significantly increased in PDAC serum. Moreover, arginine is a critical amino acid for tumor cell growth. Therefore, depletion therapy is used for patients with PDAC 38, 39. The ASS1 expression level, the rate-limiting enzyme of arginine, correlated well with the PDAC recurrence and patient survival 40. Moreover, NAC-treated patients' pancreatic tissue showed an ASS1 downregulation 40, which is in line with the current study (Fig. 3E). However, future systematic studies are required to dissect the molecular mechanism.

Also, inosine and guanosine belong to the reduced metabolites in PDAC patients' blood 36, 41, consistent with the current result. The purine metabolic pathway activation is shared among various cancers, including PDAC. For example, the purine nucleoside phosphorylase expression, which metabolizes inosine and guanosine, is upregulated in PDAC 41-43. However, PDAC's indirect effects on metabolites cannot be completely ruled out by taking the role of metabolic regulator into account. Heterogenity of intrinsic and extrinsic factors, such as dietary habits, circadian rhythms, and stress from disease, and slight differences in experimental procedures such as sample preparation and storage periods may somewhat affect metabolism. These concerns will be mitigated by large cohort studies in the future.

### The superiority of LC/ESI-MS over PESI-MS

The diagnostic PDAC system was previously reported based on the whole collection of metabolites obtained by PESI-MS in a Taiwanese cohort 32. Increasing the prediction rate in discriminating PDAC from the high-risk cohort was possible with clinical information inclusion (e.g., age and CA19-9). PESI-MS is an ambient ionization-based mass spectrometry method that surpasses the original electrospray ionization technique in its analysis rapidity and equipment simplicity. However, annotating each molecule was impossible due to technical limitations inherent to PESI-MS. Conversely, the LC/ESI-MS study successfully identified specific metabolites and demonstrated the potential for predicting PDAC at the expense of advantages unique to PESI-MS. By taking advantage of the results presented in this paper, a new version of the PESI system, the so-called PESI-MS/MS, still take over the advantage of PESI-MS as a simple diagnostic platform and additionally can identify specific molecule by collision-induced dissociation. Moreover, this retains higher throughput while achieves annotation of molecules with high accuracy. This system enables us to take two different pathways of application, namely for the clinical diagnosis on the basis of machine learning and for the basic research expertise through cutting in the specific molecular mechanisms. We will move this forward in near future.

### Potential of this system in evaluating NAC

Currently, while only curative treatment for pancreatic cancer is surgical resection, NAC has been reported to improve the prognosis and therefore gained attention in clinical settings 44, 45. However, it is not always the case that NAC is applicable to PDAC. The physical status and/or genetic or constitutional properties of patients, and non-effective cases were reported 46, 47. With this in mind, predicting the effect of NAC during treatment is valuable. The current criteria in evaluating the effects of treatment rely on the serum level of CA19-9 or imaging modalities (e.g., MRCP and CT). However, none of them is sufficient to monitor the therapeutic effect of NAC accurately. Therefore, a simple and reproducible method for assessing the antitumor effect is awaited. This study found that several metabolites differed by NAC treatment. This suggests three possibilities. First, they directly reflect PDAC malignancy, and attenuated NAC figures indicate its effectiveness. Second, NAC may change the enzymatic activity of specific metabolic pathways. Third, fundamental conditions (e.g., genetic or constitutional factors) may be different between the untreated and treated NAC cases. It is also possible that inflammation and/or cell-death-related metabolites are released from cancer with NAC treatment. Neither PM nor PL species specific to the NAC samples have been identified at least in the current study. These metabolites may be below the MS detection, or ones that were not included in the method packages. Moreover, patient outcome by life-long follow-up will help understand the exact factor that plays a pivotal role in evaluating NAC's effectiveness. Thus, future studies on large cohorts will pave the way in determining those factors.

## Supplementary Material

Supplementary figures and tables.

## Figures and Tables

**Table 1:** Patients' characteristics

		Training samples		Validation samples	
Characteristics		NC (n=20)	PDAC (n=19)	NC (n=10)	PDAC (n=10)
Age (year)	mean ± SD	71.6 ± 2.4	72.1 ± 2.0	70.7 ± 3.4	74.6 ± 2.0
Gender	male	9 (45%)	13 (68%)	4 (40%)	6 (60%)
	female	11 (55%)	6 (32%)	6 (60%)	4 (40%)
Diabetes	yes	0	8 (42%)	0	3 (30%)
	no	20	5 (26%)	10	4 (40%)
T-factor	2	-	3 (16%)	-	6 (60%)
	3	-	16 (84%)	-	4 (40%)
N-factor	0	-	6 (32%)	-	6 (60%)
	1a	-	5 (25%)	-	1 (10%)
	1b	-	0 (0%)	-	1 (10%)
	1	-	6 (32%)	-	2 (20%)
	2	-	2 (11%)	-	0 (0%)
M-factor	0	-	19 (100%)	-	10 (100%)
UICC-Stage	IB	-	0 (0%)	-	5 (50%)
	IIA	-	6 (32%)	-	2 (20%)
	IIB	-	13 (68%)	-	3 (30%)
NAC	treated	-	11 (58%)	-	9 (90%)
	untreated	-	8 (42%)	-	1 (10%)

**Table 2:** Summary of discrimination based on metabolite databases

Database	Sensitivity (True positive (TP) /PDAC samples)	Specificity (True negative (TN)/control samples)	Accuracy (TP&TN/total samples)	AUC (95% CI)
**Training samples**				
PM (91 compounds)	94.7 % (18/19)	85.0 % (17/20)	89.7 % (35/39)	0.9605 (0.8985 - 1)
PL (178 compounds)	94.7 % (18/19)	90.0 % (18/20)	92.3 % (36/39)	0.9684 (0.9168 - 1)
PM (91 compounds) PL (178 compounds)	94.7 % (18/19)	95.0 % (19/20)	94.9 % (37/39)	0.9868 (0.9581 - 1)
PM (15 compounds) PL (21 compounds)	100 % (19/19)	95.0 % (19/20)	97.4 % (38/39)	0.9974 (0.9887 - 1)
				
**Validation samples**				
PM (91 compounds) PL (178 compounds)	90 % (9/10)	100 % (10/10)	95 % (19/20)	0.97 (0.90 -1)
PM (15 compounds) PL (21 compounds)	90 % (9/10)	100 % (10/10)	95 % (19/20)	0.99 (0.96 -1)

**Table 3:** List of significantly changed metabolites identified by LC/ESI-MS

Compound name	P-value (-log10)	FC (log2)
**Primary metabolites (PM)**		
Ornithine	5.354	0.9226
Aspartic acid	4.683	1.1136
Uridine	4.658	0.7519
Glutamic acid	4.524	1.4721
Xanthine	3.124	0.8057
Hypoxanthine	2.814	1.0260
Choline	2.732	0.6664
Guanosine	2.623	-0.8194
S-Adenosylhomocysteine	2.564	1.8460
Acetylcarnitine	2.243	6.6007
Inosine	1.934	-1.1609
Arginine	1.776	0.7572
Pyruvic acid	1.674	-1.6582
Serine	1.579	0.6047
Carnosine	1.543	2.8643
		
**Phospholipids (PL)**		
PC(44:2)	4.512	-0.5883
SM(36:4)	4.250	-0.7337
PE(38:5)	4.185	0.8303
PE(38:6)	3.334	0.8950
PE(40:5)	3.187	0.7581
PE(40:7)	3.029	0.8124
PC(42:1)	2.795	-0.3537
PC(36:0)	2.687	-0.4481
PE(40:6)	2.625	0.6978
PE(38:4)	2.489	0.6261
PE(36:4)	2.394	0.8120
PC(42:2)	2.375	-0.3622
PC(42:3)	2.249	-0.4340
PE(36:6)	2.239	1.5682
PE(36:5)	2.102	0.8878
PC(32:4)	2.081	-0.5911
LPC(18:2)	2.069	-0.7811
PE(40:4)	2.032	0.6950
PE(32:1)	2.015	1.3107
PI(36:1)	1.956	-0.5903
PC(40:8)	1.923	-0.5133
PE(38:3)	1.904	0.5316
LPE(20:4)	1.897	1.7088
LPE(22:6)	1.889	0.6571
LPC(22:0)	1.879	-0.6330
LPE(18:2)	1.711	-0.7275
PC(28:0)	1.598	-0.7741
LPC(16:1)	1.558	0.2418

## Abbreviations

PDAC: Pancreatic ductal adenocarcinoma
MS: Mass spectrometry
LC: Liquid chromatography
ESI: Electrospray ionization
PESI: Probe electrospray ionization
PLS: Partial least square
ROC: Receiver operating characteristic
AUC: Area under the ROC curve
PL: Phospholipid
PM: Primary metabolite
PC: Phosphatidylcholine
PE: Phosphatidylethanolamine
SM: Sphingomyelin
NAC: Neoadjuvant chemotherapy
