# Supplementary material for: High-performance Collective Biomarker from Liquid Biopsy for Diagnosis of Pancreatic Cancer Based on Mass Spectrometry and Machine Learning
Source: J Cancer. 2021 Nov 4;12(24):7477–87. doi: 10.7150/jca.63244 (PMC8734412; doi:10.7150/jca.63244)
Supplement: Supplementary file 1 — Supplementary figures and tables. [file jcav12p7477s1.pdf]

List of Supplementary Materials

Fig. S1: Patient flow diagram.

Fig. S2: Pathway analysis of biomarkers identified in this study.

Table S1: Contents and efficacy of patients with neoadjuvant chemotherapy.

Table S2: Clinical characteristics of all control cases

Table S3: Summary of the statistical properties of metabolic pathways.

Supplementary references

Supplementary Figure 1

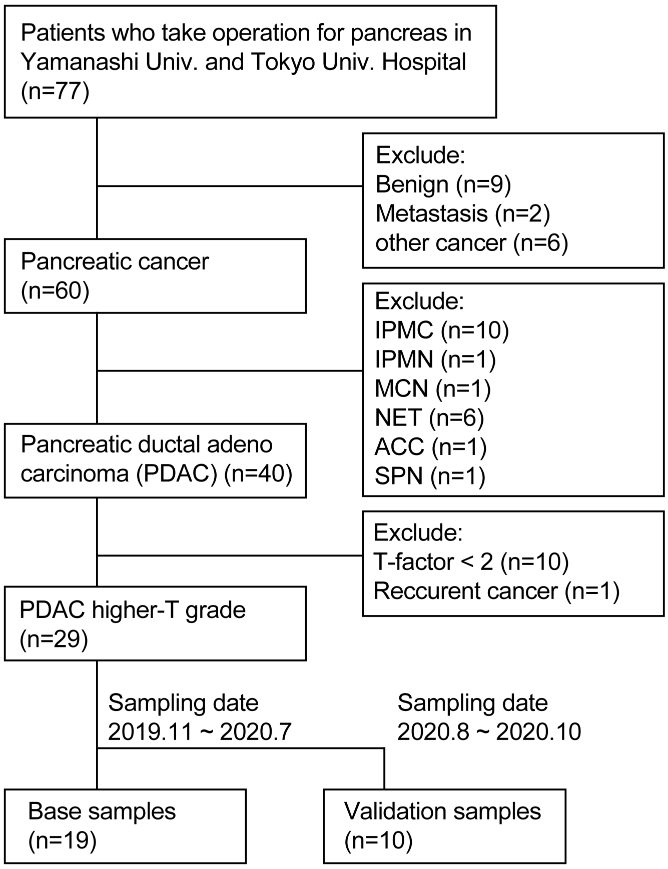

Supplementary Figure 1. Patient flow diagram.

We obtained 77 preoperative serum samples (37 from the University of Yamanashi Hospital and 40 from the University of Tokyo Hospital) from patients going through surgical resection. Of the 77 cases, 29 cases were at T2 or T3 of the TNM classification of PDAC and used for analysis in this study. Moreover, 48 cases were excluded from the analysis due to lower T grade, non-PDAC nature, or benign cases. Furthermore, 29 samples were divided into training and validation groups simply according to the admission date.

Supplementary Figure 2

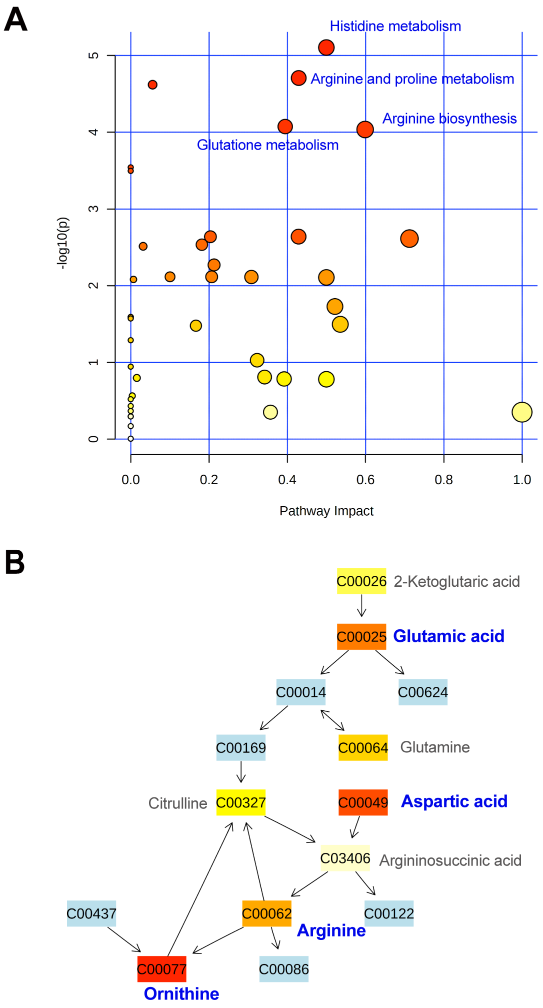

Supplementary Figure 2. Pathway analysis of biomarkers identified in this study.

A Overview of pathway analysis in terms of impact and significance of the pathway based on the database for 269 PM and PL. Significant metabolome pathways (*p*-value <0.001 and pathway impact >0.1) are labeled as the bright red plot with blue text. The statistical properties of each pathway are summarized in Table S3. **B** Overview of biosynthesis pathway for arginine with the highest impact. This pathway has four metabolites regarded as a PDAC biomarker in Fig. 3 and Table 3 highlighted by red or orange with blue text. The number in the box indicates KEGG compound ID. The metabolites with black were detected in the serum but not statistically insignificant in PDAC when compared with control. PM, primary metabolites; PL, phospholipids; PDAC, pancreatic ductal adenocarcinoma; KEGG, Kyoto Encyclopedia of Genes and Genomes.

Supplementary Table 1

Contents and efficacy of patients with neoadjuvant chemotherapy.

Variables	N=20
Resectability [1]: R/BR/UR	16/3/1
Regimen: GS/GnP	14/6
Dosing period: median (range) month	3.1 (1-14)
RECIST[2], PR/SD/PD	4/15/1
Histological response [1], grade1/grade2	17/3
Post-operative recurrence rate*	30.0 %

R, resectable; BR, borderline resectable; UR, unresectable; GS, Gemcitabine/S-1; GnP, Gemcitabine/nab-Paclitaxel; RECIST, Response evaluation criteria in solid tumors; PR, partial response; SD, stable disease; PD, progressive disease

*: median follow-up period was 9 months.

Supplementary Table 2

Clinical characteristics of all control cases

ID	collection date (yy/mm/dd)	Age (years)	gender	Diagnosis
S-01	20/03/18	62	male	Sigmoid diverticulitis
S-02	20/03/25	56	female	Hyperventilation syndrome
S-03	20/03/25	79	male	Cerebral surface vein thrombosis
S-04	20/04/01	63	female	Meniere's disease
S-05	20/04/15	83	female	Fever
S-06	20/04/29	87	female	Pseudo-gout
S-07	20/05/06	82	female	Mild heat stroke
S-08	20/05/20	81	female	Head bruise
S-09	20/05/27	81	female	Intercostal neuralgia
S-10	20/05/27	58	female	Acute gastritis
S-11	20/06/10	72	female	Fever
S-12	20/06/10	56	male	high blood pressure
S-13	20/06/24	77	male	Constipation
S-14	20/07/01	77	male	Inguinal hernia
S-15	20/07/08	72	female	Cold
S-16	20/07/15	79	male	Gastroenteritis
S-17	20/07/15	58	male	panic disorder
S-18	20/07/29	85	male	Face bruise
S-19	20/07/29	61	male	Acute gastritis
S-20	20/07/29	62	female	Epistaxis
V-01	20/08/05	86	female	Transient loss of consciousness
V-02	20/08/05	60	male	Gastro Esophageal Reflux Disease
V-03	20/08/19	73	female	Vertigo
V-04	20/08/19	58	female	Vertigo
V-05	20/09/02	79	female	Cold
V-06	20/09/09	58	male	Ureteral stone
V-07	20/09/16	61	male	Chest bruise
V-08	20/09/23	81	male	Waist bruise
V-09	20/09/23	71	female	Diarrhea
V-10	20/09/23	80	female	Pneumonia

Supplementary Table 3

Summary of the statistical properties of metabolic pathways.

Pathway name	Total	Hits	p-value	-log10(p)	FDR	Impact
Histidine metabolism	16	5	7.86E-06	5.1043	3.30.E-04	0.49999
Arginine and proline metabolism	38	9	1.96E-05	4.7068	3.36.E-04	0.42925
Glutathione metabolism	28	6	8.43E-05	4.0742	7.73.E-04	0.39476
Arginine biosynthesis	14	8	9.21E-05	4.0359	7.73.E-04	0.59898

FDR, false-discovery rate

Supplementary references

1. Isaji S. Classification of Pancreatic Carcinoma (Fourth English Edition) edited by Japan Pancreas Society. Kanehara & CO, Ltd. 2016.
2. Eisenhauer EA, Therasse P, Bogaerts J, Schwartz LH, Sargent D, Ford R, et al. New response evaluation criteria in solid tumours: revised RECIST guideline (version 1.1). European journal of cancer (Oxford, England : 1990). 2009;45(2):228-47.
